# Beyond the Lung: Geriatric Conditions Afflict Community-Dwelling Older Adults With Self-Reported Chronic Obstructive Pulmonary Disease

**DOI:** 10.3389/fmed.2022.814606

**Published:** 2022-02-14

**Authors:** Leah J. Witt, Kristen E. Wroblewski, Jayant M. Pinto, Esther Wang, Martha K. McClintock, William Dale, Steven R. White, Valerie G. Press, Megan Huisingh-Scheetz

**Affiliations:** ^1^Department of Medicine, University of California, San Francisco, San Francisco, CA, United States; ^2^Department of Public Health Sciences, The University of Chicago, Chicago, IL, United States; ^3^Department of Surgery, The University of Chicago Medicine, Chicago, IL, United States; ^4^Pritzker School of Medicine, The University of Chicago, Chicago, IL, United States; ^5^Department of Comparative Human Development, The Institute for Mind and Biology, University of Chicago, Chicago, IL, United States; ^6^Department of Supportive Care Medicine, City of Hope, Duarte, CA, United States; ^7^Department of Medicine, The University of Chicago, Chicago, IL, United States

**Keywords:** geriatrics, functional impairment, COPD–chronic obstructive pulmonary disease, polypharmacy (source: MeSH, frailty), loneliness, cognitive impairment

## Abstract

**Rationale:**

Chronic obstructive pulmonary disease (COPD) predominantly affects older adults. However, the co-morbid occurrence of geriatric conditions has been understudied.

**Objective:**

Characterize the prevalence of geriatric conditions among community-dwelling U.S. older adults with self-reported COPD.

**Methods:**

We conducted a nationally representative, cross-sectional study of 3,005 U.S. community-dwelling older adults (ages 57–85 years) from the National Social Life, Health, and Aging Project (NSHAP). We evaluated the prevalence of select geriatric conditions (multimorbidity, functional disability, impaired physical function, low physical activity, modified frailty assessment, falls, polypharmacy, and urinary incontinence) and psychosocial measures (frequency of socializing, sexual activity in the last year, loneliness, cognitive impairment, and depressive symptoms) among individuals with self-reported COPD as compared to those without. Using multivariate logistic and linear regressions, we investigated the relationships between COPD and these geriatric physical and psychosocial conditions.

**Main Results:**

Self-reported COPD prevalence was 10.7%, similar to previous epidemiological studies. Individuals with COPD had more multimorbidity [modified Charlson score 2.6 (SD 1.9) vs. 1.6 (SD 1.6)], more functional disability (58.1 vs. 29.6%; adjusted OR 3.1, 95% CI 2.3, 4.3), falls in the last year (28.4 vs. 20.8%; adjusted OR 1.4, 95% CI 1.01, 2.0), impaired physical function (75.8 vs. 56.6%; adjusted OR 2.1, 95% CI 1.1, 3.7), more frequently reported extreme low physical activity (18.7 vs. 8.1%; adjusted OR 2.3, 95% CI 1.5, 3.5) and higher frailty prevalence (16.0 vs. 2.7%; adjusted OR 6.3, 95% CI 3.0,13.0) than those without COPD. They experienced more severe polypharmacy (≥10 medications, 37.5 vs. 16.1%; adjusted OR 2.9, 95% CI 2.0, 4.2). They more frequently reported extreme social disengagement and were lonelier, but the association with social measures was eliminated when relationship status was accounted for, as those with COPD were less frequently partnered. They more frequently endorsed depressive symptoms (32.0 vs. 18.9%, adjusted OR 1.9, 95% CI 1.4, 2.7). There was no noted difference in cognitive impairment between the two populations.

**Conclusions:**

Geriatric conditions are common among community-dwelling older adults with self-reported COPD. A “beyond the lung” approach to COPD care should center on active management of geriatric conditions, potentially leading to improved COPD management, and quality of life.

## Introduction

Chronic obstructive pulmonary disease (COPD) is the third leading cause of death in the United States and fifth cause of disability in the world (1–3). COPD predominately affects older adults (4). In a 2006 study, the median global COPD pooled prevalence in people 65 years old and greater was 15%, whereas the prevalence among those 40–64 years old was 8% (5). Further, 12% of Medicare beneficiaries have COPD (6).

As people age, the development of geriatric conditions can complicate management of chronic diseases like COPD. Geriatric conditions are multifactorial disease states that transcend discrete diagnosis categories, and confer additional risk for quality of life impairment, hospitalizations, medication non-adherence and death (7–9). Frailty, a syndrome of multisystem impairment defined and assessed variably, is perhaps the best studied geriatric condition in COPD (10). Its presence has been associated with increased risk of hospitalizations and death. The prevalence of frailty in those with COPD has been estimated at almost 60%, and has been demonstrated to predict mortality better than forced expiratory volume in 1 s (11, 12). Eisner et al. found that in those with COPD, developing “non-respiratory impairment” (e.g., loss of lower extremity muscle strength) and functional limitations were associated with increased risk of disability (13). The prevalence and impact of other geriatric conditions such as multimorbidity, activities of daily living disability, physical function impairment, falls, polypharmacy, urinary incontinence, and social frailty among those with COPD have been largely understudied.

In recent years, an evolving understanding of geriatric conditions has helped paint a richer picture of the complexity of health of older people with chronic diseases. For example, research from The Health and Retirement Study, a nationally representative study of older adults, has demonstrated high rates of urinary incontinence and falls in individuals with congestive heart failure, coronary artery disease, and diabetes (14). In tandem with physical disease, social context is critical when considering health in older adults. Social frailty is an emerging concept identifying risk of losing (or loss) of valuable social resources (15). Social frailty, including social disengagement and loneliness, is more common with advancing age, increases vulnerability to catastrophic health events beyond what can be predicted by medical comorbidities alone (16), and is associated with all-cause mortality (17). In several small studies, loneliness is prevalent in those with COPD, and independently associated with more emergency room visits and reduced health perception (18–20).

The primary objective of this study is to report the prevalence of geriatric physical and psychosocial conditions among community-dwelling older adults with COPD using data from the National Social Life, Health, and Aging Project (NSHAP), a nationally representative sample. NSHAP, as compared to other longitudinal studies of older adults, is unique in its robust assessments of social health, along with other physical geriatric conditions (21). Our secondary objective was to determine whether the presence of COPD was associated with having a geriatric condition in the entire sample. We hypothesized that older adults with COPD experience accelerated physiologic aging, manifested by a much higher prevalence of geriatric conditions compared to older adults without COPD even after adjustment for demographics. Our study provides new insights into the high national rates of geriatric conditions among community dwelling people with COPD.

## Methods

### Study Population

We conducted a cross-sectional study of respondents enrolled in the first of three rounds of data collected in NSHAP. NSHAP is the first longitudinal, nationally-representative study to assess simultaneously social relationships, physical and mental health, function, and cognition in older adults (aged 57–85 at first interview) in the United States (22). This de-identified analysis was approved by the Institutional Review Board at the University of Chicago and data usage approved by the NSHAP Data Usage Agreement. All respondents provided informed consent.

### Data Collection

Round 1 was collected in 2005–2006, and enrolled 3,005 adults (1,551 women and 1,454 men) of 4,017 eligible persons, born from 1920 to 1947 (aged 57–85 at time of interview) who resided in the community (none resided in assisted living or skilled nursing facilities) (23). The unweighted response rate was 74.8% and weighted response rate was 75.5%. Data collection was comprised of three components: (1) an in-home questionnaire; (2) biomeasure collection; and (3) a self-administered leave-behind questionnaire. Potential participants were excluded in round one if they were deemed too cognitively impaired to give formal consent and/or complete the interview as determined by the field interviewer (no formal criteria).

Professional interviewers from NORC (previously known as the National Opinion Research Center) at the University of Chicago conducted the in-home assessments. Further details are available elsewhere (22, 24–28).

### Chronic Obstructive Pulmonary Disease Diagnosis

Respondents were asked the question “Has a medical doctor ever told you that you have any of the following conditions: Emphysema, chronic bronchitis, or chronic obstructive lung disease?” Responses to this question (yes/no) were used to divide the sample into comparator groups.

### Demographics

Age was calculated using date of birth and survey date. Gender (male or female), race/ethnic group (White/Caucasian, Black/African American, Hispanic/non-black, and other), smoking history, education, and relationship status were self-reported. Smoking status was categorized as “never smoker,” “former smoker,” and “current smoker,” and determined by asking respondents, “do you smoke cigarettes?” and “have you ever smoked cigarettes regularly?” Education levels were categorized as “less than high school,” “high school equivalent,” “vocational certificate,” and “bachelor's degree.” Current relationship status (currently married or in a romantic relationship) was reported as “yes” or “no”.

### Geriatric Conditions

Additional methodologic details for the geriatric conditions can be found in the Supplementary Material. Select geriatric conditions were assessed: multimorbidity (modified Charlson index score, with COPD excluded from the morbidity calculation, scale ranging from 0 to 25.5 where a 0 score indicates no co-morbid conditions and 25.5 indicates all co-morbid conditions included) (29, 30), activities of daily living (ADL) disability (see Supplementary Material), impaired physical function (timed up and go, TUG, performance time ≥ 10 s), extreme low physical activity (<once a month of moderate to vigorous activity on average), modified frailty (an adapted and abbreviated scale including exhaustion, low activity and slow TUG performance time; frailty was defined as a score of 3) (21), any fall in the last 12 months, polypharmacy (<4, 4–10, or ≥10 medications), and any urinary incontinence in last 12 months. Psychosocial measures assessed were social frailty measures: extreme social disengagement (socializing one time in the last year or less with friends or relatives), moderate social engagement (socializing several times in the last year or less with friends or relatives), and loneliness [NSHAP Felt Loneliness Measure (NFLM) ≥ 1] (28). Cognitive impairment was evaluated [moderate cognitive impairment was defined as a score of <6 on the Short Portable Mental Status Questionnaire (SPMSQ)] (31, 32). Significant depressive symptoms were assessed using the NSHAP Depressive Symptoms Measure (NDSM), with a score ≥9 demonstrating significant depressive symptoms (28). Additionally, high-risk medication usage was summed for each respondent [anti-histamines, anticholinergics, benzodiazepines, anti-psychotics, anxiolytics/sedatives, tricyclic antidepressants, muscle relaxants, anti-arrhythmic agents, cyclooxygenase (COX)-2 inhibitors, and narcotics] (33) using a medication log (34). Moderate polypharmacy was defined as taking ≥4 medications and severe polypharmacy was defined as ≥10 medications (see Supplementary Material).

### Statistical Analysis

Sample characteristics were compared among respondents with and without a self-reported doctor diagnosis of COPD. Continuous variables are presented as means with standard deviations (SD). Categorical variables are presented as percentages. *T*-tests and chi-square tests, respectively, detected significant differences between the groups.

Multivariate logistic regression models assessed the association between self-reported COPD diagnosis and each geriatric condition, adjusted for age, gender, race/ethnicity, and education. Multivariate linear regression was used to assess the association between self-reported COPD diagnosis and the modified Charlson index score. Social measures included adjustment for these demographics as well as adjustment for partner status. Odds ratios or linear regression coefficients with 95% confidence intervals (CI) are reported for all variables. *P* ≤ 0.05 were considered statistically significant. No adjustment for multiple comparisons was made. All analyses were survey weighted, accounting for the survey design, therefore reported estimates reflect the U.S. community-dwelling older adult population in 2005. Analyses were conducted in Stata 15.1 (StataCorp LLC, College Station, Texas, USA).

## Results

### Demographics

Of the 3,005 adults in Round 1, 322 respondents (10.7%) endorsed having COPD or emphysema (Table 1). Those with COPD were older (mean 69.6 years, SD 7.4 vs. 67.8 years, SD 7.7; *p* = 0.01) and more often self-identified as being white/Caucasian individuals (87.6%) as compared to the non-COPD group (79.8%). Individuals with COPD reported lower education levels (completed bachelor's degree: 19.1 vs. 25.2%), had a lower prevalence of being partnered (65.8 vs. 75.6%) and were more commonly current or former smokers (77.8 vs. 57.0%).

**Table 1:** Demographic characteristics of US older adults with and without COPD by self-report.

	**COPD** **(*n* = 322)**	**Non-COPD** **(*n* = 2,683)**	
	**Weighted % or mean (SD)**	**Weighted % or mean (SD)**	***p*-value**
TOTAL prevalence	10.7	89.3	
Age, mean years (SD)	69.6 (7.4)	67.8 (7.7)	0.01
Gender, women	55.7	51.0	0.2
**Race/Ethnicity**	(*n* = 320)	(*n* = 2,673)	0.02
White/Caucasian	87.6	79.8	
Black/African American	6.4	10.5	
Hispanic, non-black	4.2	7.2	
Other	1.8	2.6%	
**Education**			0.02
Less high school	23.6	17.9	
High school equivalent	27.8	26.8	
Vocational certificate	29.5	30.1	
Bachelor	19.1	25.2	
**Relationship status**			
Partnered	65.8	75.6	0.1
**Smoking status**	(*n* = 322)	(*n* = 2,681)	<0.0001
Current smoker	27.1	13.7	
Former smoker	50.7	43.3	
Never smoker	22.2	43.0	

### Geriatric Conditions

Older adults with self-reported COPD had more multimorbidity than those without COPD (Table 2); the average modified Charlson co-morbidity score was significantly higher (2.6, SD 1.9) as compared to the non-COPD group (1.6, SD 1.6) (*p* < 0.0001). The relationship between the modified Charlson co-morbidity score and COPD persisted after adjustment for age, race/ethnic group, gender, and education (coefficient 0.89, 95% CI 0.51, 1.27; *p* < 0.0001). They also had more asthma (34.6 vs. 7.1%), heart failure (15.4 vs. 7.4%), history of myocardial infarction (19.5 vs. 10.7%), history of cerebral vascular events/stroke (14.7 vs. 7.3%), and arthritis (68.4 vs. 49.5%).

**Table 2:** Prevalence of multimorbidity among US older adults with and without COPD by self-report.

	**COPD** **(*n* = 322)**	**Non-COPD** **(*n* = 2,683)**	
Modified Charlson^*^, mean (SD)	2.6 (1.9)	1.6 (1.6)	<0.0001
**Select conditions**			
Asthma	34.6%	7.1%	<0.0001
Arthritis	68.4%	49.5%	<0.0001
History of stroke	14.7%	7.3%	<0.0001
Heart failure	15.4%	7.4%	0.0009
History of MI	19.5%	10.7%	0.003
Diabetes	22.7%	19.4%	0.3
Cancer (ever had)	12.5%	11.4%	0.6

*COPD, chronic obstructive pulmonary disease; MI, myocardial infarction*.

* *Modified Charlson co-morbidity index: as previously described in the NSHAP data set based on the original index of 19 weighted conditions; co-morbidities were added with varying weights as follows: 1 point assigned to history of myocardial infarction, gastric ulcer disease, congestive heart failure, peripheral vascular disease, arthritis, dementia, asthma, and stroke; 1.5 points assigned to diabetes, 2 points assigned to liver disease, leukemia, lymphoma, renal disease, and cancer history; and 6 points assigned to metastatic cancer. COPD was removed from the score. Possible score ranged from 0 to 25.5 where a 0 score indicates no co-morbid conditions and 25.5 indicates all co-morbid conditions included*.

**Figure 1:**
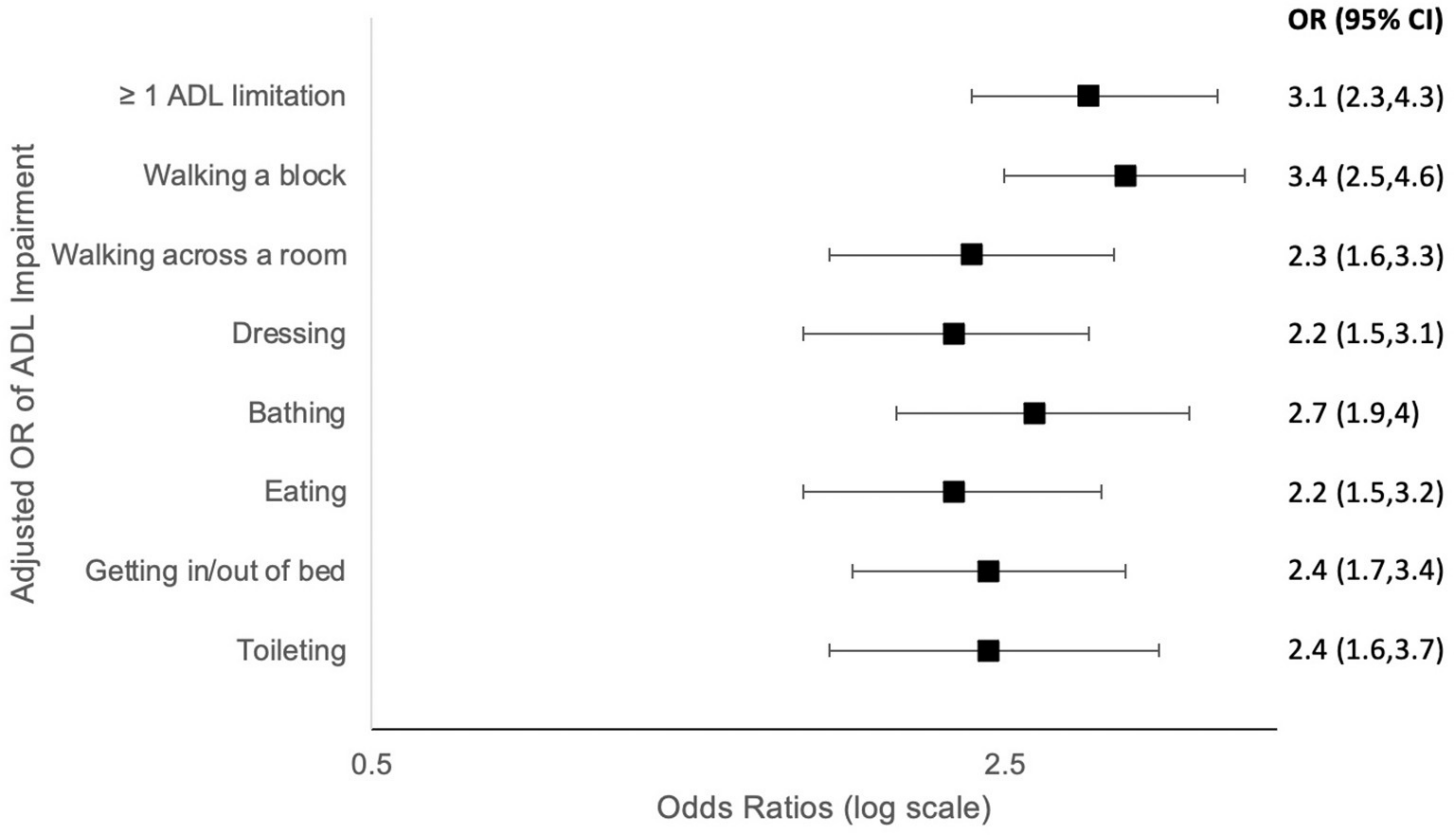
Forest plots (odds ratios with 95% confidence intervals) based on Multivariate Logistic regression models comparing activities of daily living (ADL) impairment among US older adults with vs. without COPD by self-report. OR, odds ratio; ADL, activities of daily living; Adjusted model: adjusted for age, gender, race/ethnic group, and education.

Older adults with self-reported COPD had higher rates of at least one ADL disability (58.1 vs. 29.6%, adjusted model OR 3.1, 95% CI 2.3, 4.3; *p* < 0.0001; Table 3). They reported more difficulty performing every reported ADL (walking a block, walking across a room, dressing, bathing, eating, bed mobility, and toileting) (Figure 1). The most profound impairment was difficulty walking a block compared to those without COPD (OR 3.4, 95% CI 2.5, 4.6; *p* < 0.0001).

**Table 3:** Unadjusted and adjusted multivariate logistic regression models comparing the prevalence of geriatric conditions among US older adults with and without COPD by self-report.

	**COPD %**	**Non-COPD %**	**Unadjusted model**	**Adjusted model**
			**OR (95% CI)**	**OR (95% CI)**
**Physical measures**				
At least 1 ADL limitation	58.1	29.6	3.3 (2.4, 4.5)	3.1 (2.3, 4.3)
Slow gait (TUG) speed (≥10 s)	75.8	56.6	2.4 (1.4, 4.1)	2.1 (1.1, 3.7)
Extreme low physical activity (< once a month)	18.7	8.1	2.6 (1.8, 3.7)	2.3 (1.5, 3.5)
Frail (abbreviated scale)	16.0	2.7	6.8 (3.5, 13.2)	6.3 (3.0, 13.0)
Fall (in last 12 months)	28.4	20.8	1.5 (1.1, 2.1)	1.4 (1.0, 2.0)
Urinary incontinence (in last 12 months)	53.9	39.6	1.8 (1.4, 2.3)	1.7 (1.3, 2.1)
**Psychosocial measures**				
Extreme social disengagement^*^ (once a year or less)	4.5	2.1	2.2 (1.2, 4.0)	0.7 (0.1, 4.8)
Moderate social disengagement^*^ (several times a year or less)	23.1	22.7	1.0 (0.8, 1.4)	0.8 (0.5, 1.5)
No sex (in last year)^*^	60.9	42.8	2.1 (1.5, 2.8)	1.5 (0.7, 2.9)
Loneliness^*^ (NFLM ≥ 1)	57.7	42.1	1.9 (1.4, 2.5)	1.2 (0.7, 2.2)
Moderate cognitive impairment (SPMSQ <6)	12.9	17.6	0.7 (0.3, 2.1)	0.6 (0.2, 1.9)
Frequent depressive symptoms (NDSM ≥ 9)	32.0	18.9	2.0 (1.5, 2.8)	1.9 (1.4, 2.7)

*OR, odds ratio; CI, confidence interval; ADL, activity of daily living; TUG, timed up-and-go; NFLM, NSHAP felt loneliness measure; NDSM, NSHAP depressive symptoms measure*.

*Adjusted model: adjusted for age, gender, race/ethnic group, and education*.

* *Adjusted model also included relationship status*.

Older adults with self-reported COPD had more frequently impaired physical function as measured by a slow TUG test (≥10 s): 75.8 vs. 56.6%, adjusted OR 2.1, 95% CI 1.1, 3.7; *p* < 0.02; Table 3). They also reported more extreme physical inactivity (18.7 vs. 8.1%, adjusted OR 2.3, 95% CI 1.5, 3.5; *p* < 0.0001). Modified physical frailty (as identified by presence of 3 criteria using an adapted and modified 3-point scale) was more common: 16.0 vs. 2.7% (adjusted OR 6.3, 95% CI 3.0, 13.0; *p* < 0.0001). They reported falling in the last year more frequently than those without COPD (28.4 vs. 20.5%, adjusted OR 1.4, 95% CI 1.01, 2.0; *p* = 0.04; Table 3). Urinary incontinence was highly prevalent in older adults with COPD (53.9 vs. 39.6%, adjusted OR 1.7, 95% CI 1.3, 2.1; *p* < 0.0001; Table 3).

Older adults with self-reported COPD had significantly more moderate polypharmacy (≥4 medications) (80.6 vs. 58.4%, adjusted OR 2.7, 95% CI 2.0, 3.8; *p* < 0.0001) and severe polypharmacy (≥10 medications) (37.5 vs. 16.1%, adjusted OR 2.9, 95% CI 2.0, 4.2; *p* < 0.0001; Table 4). Respondents in the COPD group were found to be taking many more high-risk medications, such as anti-histamines, benzodiazepines, and narcotics (Table 4).

**Table 4:** Polypharmacy and high-risk medications among US older adults with and without COPD by self-report.

	**COPD %**	**Non- COPD %**	**Unadjusted**	**Adjusted**
			**OR (95% CI)**	**OR (95% CI)**
**Polypharmacy**				
Moderate (≥4 medications)	80.6	58.4	3.0 (2.1, 4.1)	2.7 (2.0, 3.8)
Severe (≥10 medications)	37.5	16.1	3.1 (2.2, 4.5)	2.9 (2.0, 4.2)
**High-Risk meds**				
Anti-histamines	16.8	6.0	3.2 (2.2, 4.6)	3.4 (2.2, 5.1)
Anticholinergic/	4.0	1.3	3.2 (1.4, 7.5)	2.7 (1.2, 6.4)
anti-spasmodic				
Benzodiazepines	11.1	5.4	2.2 (1.4, 3.5)	1.9 (1.2, 3.0)
Anti-psychotics	2.9	1.3	2.2 (1.1, 4.5)	1.9 (0.99, 3.7)
Anxiolytic/Sedatives	15.3	8.0	2.1 (1.4, 3.1)	1.9 (1.3, 2.8)
Tricyclic anti-depressant	4.8	2.0	2.6 (1.2, 5.7)	2.4 (1.1, 5.2)
Muscle relaxants	4.9	1.6	3.1 (1.3, 7.1)	3.4 (1.4, 8.0)
Anti-arrhythmics	3.3	1.2	2.9 (1.4, 6.0)	2.6 (1.2, 5.7)
COX-2 inhibitors	4.1	2.0	2.0 (1.1, 4.0)	1.9 (0.98, 3.9)
Narcotics	9.3	4.5	2.2 (1.1, 4.1)	2.0 (1.1, 3.8)

*COPD, chronic obstructive pulmonary disease; OR, odds ratio; CI, confidence interval; COX-2, cyclooxygenase-2*.

*Adjusted model: adjusted for age, gender, race/ethnic group, and education*.

Community-dwelling older U.S. adults with self-reported COPD had more extreme social disengagement, as assessed by higher frequency of socializing less than once a year with family and friends (4.5 vs. 2.1%, unadjusted OR 2.2, 95% CI 1.2, 4.0; *p* = 0.01, adjusted OR 0.7, 95% CI 0.1, 4.8, *p* = 0.7). Moderate social disengagement was not significantly different between the COPD and non-COPD groups (23.1 vs. 22.7%, unadjusted OR 1.0, 95% CI 0.8, 1.4, *p* = 0.18, adjusted OR 0.8, 95% CI 0.5, 1.5; *p* = 0.5). They also had higher rates of sexual inactivity in the last year (60.9 vs. 42.8%, unadjusted OR 2.1, 95% CI 1.5, 2.8; *p* < 0.0001, adjusted OR 1.5, 95% CI 0.7, 2.9, *p* = 0.3). They were also lonelier (57.7 vs. 42.1%, unadjusted OR 1.9, 95% CI 1.4, 2.5; *p* < 0.0001, adjusted OR 1.2, 95% CI 0.7, 2.2; *p* = 0.5; Table 3). These differences were largely due to partnership status, as the significance of these associations diminished in the models which adjusted for relationship status.

The SPMSQ cognitive assessment did not uncover significant differences in cognitive impairment in those with self-reported COPD compared to those without (12.9 vs. 17.6%, adjusted OR 0.6, 95% CI 0.2, 1.9, *p* = 0.4). Those with COPD more frequently reported depressive symptoms by the NSHAP Depressive Symptoms Measure (32.0 vs. 18.9%, adjusted OR 1.9, 95% CI 1.4, 2.7; *p* < 0.0001).

## Discussion

Our study establishes that COPD is frequently co-prevalent with multiple, non-respiratory domains of age-related vulnerability requiring complex and coordinated interdisciplinary care and specialized geriatrics training and knowledge (35). Our findings also demonstrate compelling evidence of social frailty among older U.S. adults with COPD living at home, likely related to the significant difference in partnership status between the groups.

In the United States, community-dwelling older adults with COPD are disproportionately afflicted with geriatric conditions that reflect worse global physical and social health. Previous work has demonstrated a high rate of disability and social disengagement among community dwelling older adults with COPD (36). Our findings confirm this and expand upon other physical and social burdens of COPD, with new information about the high burden of multimorbidity, functional disability, impaired physical function (by slow TUG performance time), low physical activity, falls, polypharmacy, urinary incontinence, depressive symptoms, and both physical and social frailty in a nationally representative community dwelling population with COPD. These findings make clear the larger ecological burden of COPD on older Americans.

The U.S. health system siloes disease management by organ system and subspecialty. This has led to traditional clinical assessments of COPD severity that miss the mark and focus narrowly on COPD-specific issues such as exacerbations, lung function and dyspnea. Our data show that clinicians caring for people with COPD need to consider larger issues of social health and ecology in the care of these patients.

Social health is one critical pillar of wellbeing that often is not captured by traditional organ-centric medical models of health (16). Our findings of social disengagement are of clear importance in the broader care of patients with COPD. As one example, we found that these patients are lonelier, have more extreme social disengagement, and pursue less frequent sexual activity. Interestingly, these effects appears to be primarily related to not having a partner as the significant effect was eliminated once the analyses were adjusted for relationship status. This finding highlights that the social history may be useful to understand the wider burden of COPD in this population and the common lack of a strong social infrastructure to assist with disease management. This finding has clinical relevance because loneliness has been demonstrated to be associated with more emergency room visits and reduced health perception in people with COPD (20). Compared to previous studies on the prevalence of loneliness in which estimates ranged from 25 to 29%, both the COPD and non-COPD populations were lonelier (37). Further, sexual relationships and dysfunction have been demonstrated previously to be common among those with COPD and have an underappreciated impact on quality of life (38–40). Identifying loneliness and social disengagement in patients with COPD may allow clinicians and other caregivers to develop strategies to improve engagement, aided by recommendations from interprofessional team members such as social workers and physical therapists.

A high prevalence of depressive symptoms was demonstrated in the self-reported COPD population, which has been reported previously (41). Depressive symptoms in COPD has been linked to increased acute exacerbations and mortality (42, 43). Frequent assessments for depression with in-office tools such as the PHQ-2 and PHQ-9 are critical, and mental health support and referrals should be pursued by primary care providers and specialty teams caring for patients with COPD and depressive symptoms.

Among older U.S. adults with COPD, there were high rates of ADL disability and physical function impairment along with physical frailty by a modified index. These individuals also were less physically active and suffered more falls. Disability and impaired physical function lead to a decline in independent living, sometimes in catastrophic situations (e.g., following hip fracture), and people who maintain mobility have higher late-life function and quality of life (44–46). Those with COPD are particularly vulnerable due to breathlessness and loss of muscle mass (sarcopenia) (47–49). We propose incorporating simple geriatric assessments into the routine care of people with COPD. Such assessments are likely to uncover unmet need for assistive devices (e.g., walkers and canes, durable medical equipment (e.g., shower chairs), strength training or consideration for additional care (e.g., disability parking placards, in-home caregiving) (50).

Polypharmacy increases mortality in the general older adult population (51). We found significant polypharmacy in patients with COPD as well as increased use of potentially inappropriate and high-risk medications. Measures to identify and limit polypharmacy are especially important in older adults with COPD to limit potentially harmful side effects. Several medications in the high-risk categories for these patients include narcotics and benzodiazepines that may depress respiration. Polypharmacy may be related to their higher rates of multimorbidity which often leads to increased clinical encounters, including subspecialty visits and hospitalizations, and subsequent medication prescribing, as has been demonstrated in other contexts (52, 53). Previous work has demonstrated limited understanding of such geriatric issues in subspecialty and general medical trainees (54) which we hypothesize carries forward to long-term practice patterns that result (in part) in polypharmacy. Pulmonary specialty training should include of geriatrics education, in which geriatric conditions, polypharmacy and high-risk medications are learned, as such knowledge may equip specialists with tools to manage COPD more optimally. The impact of this training will require further study.

Urinary incontinence is a highly prevalent geriatric comorbidity that impairs quality of life and leads to falls (55, 56). We found that urinary incontinence was common in both groups, but older adults with COPD had significantly more urinary incontinence in the prior year as compared to those without COPD. The urinary incontinence definition used in NSHAP was very inclusive as it captured any related symptoms regardless of frequency in the last year. This definition may have included those with rare symptoms. We hypothesize that contributors to urinary incontinence in COPD include frequent coughing, medication side effects, generalized sarcopenia that includes pelvic floor muscles, and decreased ability to ambulate to the bathroom and thus functional incontinence. Screening of and treatment for urine incontinence, including non-pharmacologic options (e.g., pessaries, pelvic floor physical therapy), should be offered to patients with COPD when identified and can greatly improve quality of life.

Surprisingly, higher rates of cognitive impairment were not seen in the NSHAP COPD population, which differs from many previous studies (57, 58). A possible cause for this finding is the low-sensitivity of the SPMSQ cognitive assessment tool used in Round 1 of NSHAP data collection, which is unable to detect early, more subtle cognitive changes. This assessment tool was replaced by a survey-adapted Montreal Cognitive Assessment (MoCA-SA) in subsequent rounds. Future studies will need to assess the burden of cognitive impairment among NSHAP's self-reported COPD population using this more sensitive screening tool. Another possible cause of this finding is that potential participants were excluded in Round 1 if they were too cognitively impaired to give formal consent, which likely excluded participants with more severe cognitive impairment.

A strength of our study is the generalizability of our findings which are based on a nationally representative study of older U.S. adults, with robust assessments of social function and context and simultaneous measures of physical health. Our study is limited by the lack of spirometric data in NSHAP to verify obstructive lung disease diagnosis or stratify outcomes by COPD severity. Additionally, we suspect there may be overlap with other airway disease in some individuals who self-reported asthma but not COPD; this is a diagnostic challenge in the field more generally. Because COPD is a clinical diagnosis that must include assessment of symptoms and exposures along with spirometry, we caution that using spirometry alone to determine case definition of COPD would also have challenges. For example, age-related lung function changes may cause an obstructive pattern and could lead to inclusion of participants without COPD. We note that the prevalence of COPD by self-report in the NSHAP population is consistent with previously epidemiologic reports based on rigorous criteria (1). Self-reported disease data may also have affected the accuracy of the modified Charlson comorbidity index (for example the high reported co-prevalence of asthma and COPD suggest that participants may have mischaracterized their lung disease in reporting). However, this method of reporting is common, as the US Centers for Disease Control assesses COPD prevalence via self-report via the Behavioral Risk Factor Surveillance System telephone survey (59).

Another potential limitation of our study is the significant age difference between the COPD and non-COPD participant groups; those with COPD were almost 2 years older than the non-COPD group. While our analyses were adjusted for age, there may be unaccounted for age effects that influenced the findings of increased geriatric conditions in this group. Our frailty assessment was adapted and not validated, given absence of weight loss and hand grip data in Round 1, so this should be interpreted with caution. Our frailty prevalence was lower than expected compared to national rates in the National Health and Aging Trends Study, which used validated scales and found a prevalence of 15% (95% CI: 14, 16%) in the older non-nursing home population (60). Finally, NSHAP lacks COPD-specific quality of life questions to assess for cough and breathlessness which is another limitation. This information is now used to classify severity of COPD and may be linked to deteriorating physical function and social disengagement (61).

Our findings suggest that a geriatric-focused approach to COPD care could reap significant benefits for affected individuals. Unfortunately, geriatricians are in short-supply and cannot practically care for all patients that could benefit. In 2018, there were about two pulmonologists to every geriatrician in the U.S. (14,899 vs. 7,290), so it is imperative that health systems innovate in order to extend age-friendly care to those that need it (62, 63). The field of geriatric oncology has been a pioneer in geriatric-subspecialty care and have endorsed comprehensive geriatric assessments (CGAs) in older patients with cancer (64). In practice, execution of these geriatric evaluations range from sponsoring embedded consulting geriatricians to perform CGAs for high-risk patients, training interprofessional team members to deliver simple screening assessments, or empowering subspecialists to become dually trained in geriatrics and their intended subspecialty (65). All of these models are possible in ambulatory pulmonary care.

When social or physical frailty are identified, management recommendations should include referrals to interprofessional and multidisciplinary team members, which is a core tenet of age-friendly care. For example, social workers can offer support, counsel, and referrals to social engagement and caregiving resources, physical therapists can help address sarcopenia and frailty, behavioral health specialists can provide counseling and treatment for depressive symptoms, and medical assistants, nursing staff, respiratory therapists and pharmacists can ensure medication lists are up to date and patients are trained in correct inhaler device use. Well-informed providers and clinics can and should assess for unmet medical equipment needs to reduce the mismatch between an individual's environment and their physical capabilities (e.g., shower chairs, raised toilet seats, grab bars, canes, walkers, and disability parking placards) (50). Finally, pulmonary specialty training should include geriatrics education, and providers should enter independent practice armed with specialization in age-friendly COPD care (65, 66). This multi-pronged, “beyond the lung” approach is likely to lead to improved COPD management and quality of life for this population.

## Conclusion

Geriatric conditions disproportionately afflict community-dwelling older adults with COPD. The presence of multiple domains of vulnerability directly impact COPD management, therefore COPD care requires a geriatric lens. A “beyond the lung” approach to COPD care should be prioritized by the siloed U.S health system, health care organizations and individual providers, which will potentially lead to improved quality of life and COPD management for affected individuals.

## Data Availability Statement

The raw data supporting the conclusions of this article will be made available by the authors, without undue reservation. Round 1 data is available through the National Archive of Computerized Data on Aging (NACDA): https://www.icpsr.umich.edu/web/pages/NACDA/nshap.html.

## Ethics Statement

The studies involving human participants were reviewed and approved by Institutional Review Board at the University of Chicago. The patients/participants provided their written informed consent to participate in this study.

## Author Contributions

LW, KW, JP, EW, MM, WD, SW, VP, and MH-S made substantial contributions to the conception and design of the work. LW and MH-S wrote the first draft of the manuscript. All authors listed above agree to be accountable for all aspects of the work in ensuring that questions related to the accuracy or integrity of any part of the work are appropriately investigated and resolved and substantial contributions to the acquisition, analysis, or interpretation of data for the work. All authors contributed to the article and approved the submitted version.

## Funding

The National Social Life, Health, and Aging Project was supported by the National Institutes of Health, including the National Institute on Aging, the Office of Women's Health Research, the Office of AIDS Research, and the Office of Behavioral and Social Sciences Research (Nos. R01AG021487, R01AG043538-06, and R01AG048511-06). LW funding support: NIH funded Research Training in Respiratory Biology grant at the University of Chicago (No. T32 HL007605). The project described was supported by Grant No. K01HP334460100 from the Health Resources and Services Administration (HRSA), an operating division of the U.S. Department of Health and Human Services. VP reports receiving funding from the NIH (HL146644), AHRQ (R01HS027804-01A1), American Lung Association (Innovation Award). Funding support for MH-S was provided by NIH NIA 1K23AG049106. SW reports funding from NIH (Nos. UG1-HL139125, R34 HL136991, R01 HL104068, and T32 HL007605). JP reports funding from NIA (No. AG067497).

## Author Disclaimer

The contents are solely the responsibility of the authors and do not necessarily represent the official views of the Health Re-sources and Services Administration or the U.S. Department of Health and Human Services.

## Conflict of Interest

VP reports receiving consultant fees from Vizient and Humana. SW reports receiving consulting and speaking fees from Regeneron, Inc., Astra-Zeneca, Inc., Sanofi Genzyme, Inc., and the CHEST Foundation. JP reports receiving speaker's/consulting fees from Regeneron, Inc., Sanofi Genzyme, Inc., and Optinose, Inc. JP also serves as site investigator for clinical trial supported by Optinose, Inc., Connect Pharma, Inc., Regeneron, Inc., and Sanofi-Genzyme, Inc. The remaining authors declare that the research was conducted in the absence of any commercial or financial relationships that could be construed as a potential conflict of interest.

## Publisher's Note

All claims expressed in this article are solely those of the authors and do not necessarily represent those of their affiliated organizations, or those of the publisher, the editors and the reviewers. Any product that may be evaluated in this article, or claim that may be made by its manufacturer, is not guaranteed or endorsed by the publisher.

## References

[1] Adeloye D Chua S Lee C Basquill C Papana A Theodoratou E. Global and regional estimates of COPD prevalence: systematic review and meta-analysis. J Glob Health. (2015) 5:020415. 10.7189/jogh.05.020415 26755942 PMC4693508

[2] Ford ES. Trends in mortality from COPD among adults in the United States. Chest. (2015) 148:962–70. 10.1378/chest.14-2311 25411775 PMC4587987

[3] Vos T Flaxman AD Naghavi M Lozano R Michaud C Ezzati M. Years lived with disability (YLDs) for 1160 sequelae of 289 diseases and injuries 1990-2010: a systematic analysis for the global burden of disease study 2010. Lancet. (2012) 380:2163–96. 10.1016/S0140-6736(12)61729-2 23245607 PMC6350784

[4] Tilert T Dillon C Paulose-Ram R Hnizdo E Doney B. Estimating the U.S. prevalence of chronic obstructive pulmonary disease using pre- and post-bronchodilator spirometry: the national health and nutrition examination survey (NHANES) 2007-2010. Respir Res. (2013) 14:103. 10.1186/1465-9921-14-103 24107140 PMC3854606

[5] Halbert RJ Natoli JL Gano A Badamgarav E Buist AS Mannino DM. Global burden of COPD: systematic review and meta-analysis. Euro Respir J. (2006) 28:523–32. 10.1183/09031936.06.00124605 16611654

[6] Centers for Medicare and Medicaid Services. Chronic Conditions Among Medicare Beneficiaries, Chartbook, 2012 edition. Baltimore, MD: Centers for Medicare and Medicaid Services (2012).

[7] Inouye SK Studenski S Tinetti ME Kuchel GA. Geriatric syndromes: clinical, research and policy implications of a core geriatric concept. J Am Geriatr Soc. (2007) 55:780–91. 10.1111/j.1532-5415.2007.01156.x 17493201 PMC2409147

[8] Wang SY Shamliyan TA Talley KMC Ramakrishnan R Kane RL. Not just specific diseases: systematic review of the association of geriatric syndromes with hospitalization or nursing home admission. Arch Gerontol Geriatr. (2013) 57:16–26. 10.1016/j.archger.2013.03.007 23578847

[9] Kane RL Shamliyan T Talley K Pacala J. The association between geriatric syndromes and survival. J Am Geriatr Soc. (2012) 60:896–904. 10.1111/j.1532-5415.2012.03942.x 22568483

[10] Fried LP Tangen CM Walston J Newman AB Hirsch C Gottdiener J. Frailty in older adults evidence for a phenotype. J Gerontol Ser A Biol Sci Med Sci. (2001) 56:M146–57. 10.1093/gerona/56.3.M146 11253156

[11] Park SK Richardson CR Holleman RG Larson JL. Frailty in people with COPD, using the national health and nutrition evaluation survey dataset (2003–2006). Heart Lung. (2013) 42:163–70. 10.1016/j.hrtlng.2012.07.004 23535142 PMC4020241

[12] Lahousse L Ziere G Verlinden VJ Zillikens MC Uitterlinden AG Rivadeneira F. Risk of frailty in elderly with COPD: a population-based study. J Gerontol Ser A Biol Sci Med Sci. (2016) 71:689–95. 10.1093/gerona/glv154 26355016

[13] Eisner MD Iribarren C Blanc PD Yelin EH Ackerson L Byl N. Development of disability in chronic obstructive pulmonary disease: beyond lung function. Thorax. (2011) 66:108–14. 10.1136/thx.2010.137661 21047868 PMC3111223

[14] Lee PG Cigolle C Blaum C. The co-occurrence of chronic diseases and geriatric syndromes: the health and retirement study. J Am Geriatr Soc. (2009) 57:511–6. 10.1111/j.1532-5415.2008.02150.x 19187416

[15] Bunt S Steverink N Olthof J van der Schans CP Hobbelen JSM. Social frailty in older adults: a scoping review. Eur J Ageing. (2017) 14:323–34. 10.1007/s10433-017-0414-7 28936141 PMC5587459

[16] McClintock MK Dale W Laumann EO Waite L. Empirical redefinition of comprehensive health and well-being in the older adults of the United States. Proc Natl Acad Sci USA. (2016) 113:E3071–80. 10.1073/pnas.1514968113 27185911 PMC4896706

[17] Steptoe A Shankar A Demakakos P Wardle J. Social isolation, loneliness, and all-cause mortality in older men and women. Proc Natl Acad Sci USA. (2013) 110:5797–801. 10.1073/pnas.1219686110 23530191 PMC3625264

[18] Petitte T Mallow J Barnes E Petrone A Barr T Theeke L. A systematic review of loneliness and common chronic physical conditions in adults. Open Psychol J. (2015) 8 (Suppl. 2):113–32. 10.2174/1874350101508010113 26550060 PMC4636039

[19] Kara M Mirici A. Loneliness, depression, and social support of Turkish patients with chronic obstructive pulmonary disease and their spouses. J Nurs Scholarsh. (2004) 36:331–6. 10.1111/j.1547-5069.2004.04060.x 15636413

[20] Marty PK Novotny P Benzo RP. Loneliness and ED visits in chronic obstructive pulmonary disease. Mayo Clin Proc Innov Qual Outcomes. (2019) 3:350–7. 10.1016/j.mayocpiqo.2019.05.002 31485574 PMC6713837

[21] Huisingh-Scheetz M Kocherginsky M Schumm PL Engelman M McClintock MK Dale W. Geriatric syndromes and functional status in NSHAP: rationale, measurement, and preliminary findings. J Gerontol B Psychol Sci Soc Sci. (2014) 69 (Suppl. 2):S177–90. 10.1093/geronb/gbu091 25360019 PMC4303102

[22] Smith S Jaszczak A Graber J Lundeen K Leitsch S Wargo E. Instrument development, study design implementation, and survey conduct for the national social life, health, and aging project. J Gerontol B Psychol Sci Soc Sci. (2009) 64B (Suppl 1):i20–9. 10.1093/geronb/gbn013 19357076 PMC2800812

[23] Waite LJ Laumann EO Levinson W Lindau ST O'Muircheartaigh CA. National Social Life, Health, and Aging Project (NSHAP): Wave 1. Ann Arbor, MI: Inter-University Consortium for Political and Social Research (2014).

[24] Drum ML Shiovitz-Ezra S Gaumer E Lindau ST. Assessment of smoking behaviors and alcohol use in the national social life, health, and aging project. J Gerontol B Psychol Sci Soc Sci. (2009) 64 (Suppl. 1):i119–30. 10.1093/geronb/gbn017 19181686 PMC2763525

[25] Suzman R. The national social life, health, and aging project: an introduction. J Gerontol B Psychol Sci Soc Sci. (2009) 64B (Suppl. 1):i5–11. 10.1093/geronb/gbp078 19837963 PMC2763520

[26] Waite LJ Laumann EO Das A Schumm LP. Sexuality: measures of partnerships, practices, attitudes, and problems in the national social life, health, and aging study. J Gerontol B Psychol Sci Soc Sci. (2009) 64B (Suppl. 1):i56–66. 10.1093/geronb/gbp038 19497930 PMC2763521

[27] Jaszczak A Lundeen K Smith S. Using nonmedically trained interviewers to collect biomeasures in a national in-home survey. Field methods. (2009) 21:26–48. 10.1177/1525822X08323988 21796261 PMC3143069

[28] Payne C Hedberg EC Kozloski M Dale W McClintock MK. Using and interpreting mental health measures in the national social life, health, and aging project. J Gerontol B Psychol Sci Soc Sci. (2014) 69 (Suppl. 2):S99–116. 10.1093/geronb/gbu100 25360028 PMC4303090

[29] Charlson ME Pompei P Ales KL MacKenzie CR. A new method of classifying prognostic comorbidity in longitudinal studies: development and validation. J Chronic Dis. (1987) 40:373–83. 10.1016/0021-9681(87)90171-8 3558716

[30] Vasilopoulos T Kotwal A Huisingh-Scheetz MJ Waite LJ McClintock MK Dale W. Comorbidity and chronic conditions in the national social life, health and aging project (NSHAP), wave 2. J Gerontol Ser B. (2014) 69:S154–65. 10.1093/geronb/gbu025 25360017 PMC4303089

[31] Pfeiffer E. A short portable mental status questionnaire for the assessment of organic brain deficit in elderly patients. J Am Geriatr Soc. (1975) 23:433–41. 10.1111/j.1532-5415.1975.tb00927.x 1159263

[32] Laumann EO Leitsch SA Waite LJ. Elder mistreatment in the united states: prevalence estimates from a nationally representative study. J Gerontol Ser B. (2008) 63:S248–54. 10.1093/geronb/63.4.S248 18689774 PMC2756833

[33] By the 2019 American Geriatrics Society Beers Criteria® Update Expert Panel. American geriatrics society 2019 updated AGS beers criteria® for potentially inappropriate medication use in older adults. J Am Geriatr Soc. (2019) 67:674–94. 10.1111/jgs.15767 30693946

[34] Qato DM Schumm LP Johnson M Mihai A Lindau ST. Medication data collection and coding in a home-based survey of older adults. J Gerontol B Psychol Sci Soc Sci. (2009) 64B (Suppl. 1):i86–93. 10.1093/geronb/gbp036 19491196 PMC2763517

[35] Fried TR Vaz Fragoso CA Rabow MW. Caring for the older person with chronic obstructive pulmonary disease. JAMA. (2012) 308:1254–63. 10.1001/jama.2012.12422 23011715 PMC3815613

[36] Liu Y Croft JB Anderson LA Wheaton AG Presley-Cantrell LR Ford ES. The association of chronic obstructive pulmonary disease, disability, engagement in social activities, and mortality among US adults aged 70 years or older, 1994–2006. Int J Chron Obstruct Pulmon Dis. (2014) 9:75–83. 10.2147/COPD.S53676 24477269 PMC3896280

[37] Ong AD Uchino BN Wethington E. Loneliness and health in older adults: a mini-review and synthesis. Gerontology. (2016) 62:443–9. 10.1159/000441651 26539997 PMC6162046

[38] Collins EG Halabi S Langston M Schnell T Tobin MJ Laghi F. Sexual dysfunction in men with COPD: impact on quality of life and survival. Lung. (2012) 190:545–56. 10.1007/s00408-012-9398-4 22752718

[39] Zysman M Rubenstein J Le Guillou F Colson RM Pochulu C Grassion L. COPD burden on sexual well-being. Respir Res. (2020) 21:311. 10.1186/s12931-020-01572-0 33238993 PMC7687801

[40] Kaptein AA van Klink RC de Kok F Scharloo M Snoei L Broadbent E. Sexuality in patients with asthma and COPD. Respiratory Medicine. (2008) 102:198–204. 10.1016/j.rmed.2007.09.012 17996435

[41] Connolly MJ Yohannes AM. The impact of depression in older patients with chronic obstructive pulmonary disease and asthma. Maturitas. (2016) 92:9–14. 10.1016/j.maturitas.2016.07.005 27621232

[42] Jennings JH DiGiovine B Obeid D Frank C. The association between depressive symptoms and acute exacerbations of COPD. Lung. (2009) 187:128–35. 10.1007/s00408-009-9135-9 19198940

[43] de Voogd JN Wempe JB Koëter GH Postema K van Sonderen E Ranchor AV. Depressive symptoms as predictors of mortality in patients with COPD. Chest. (2009) 135:619–25. 10.1378/chest.08-0078 19029432

[44] Gill TM. Disentangling the disabling process: insights from the precipitating events project. Gerontologist. (2014) 54:533–49. 10.1093/geront/gnu067 25035454 PMC4155452

[45] Vaughan L Leng X La Monte MJ Tindle HA Cochrane BB Shumaker SA. Functional independence in late-life: maintaining physical functioning in older adulthood predicts daily life function after age 80. J Gerontol A Biol Sci Med Sci. (2016) 71 (Suppl. 1):S79–86. 10.1093/gerona/glv061 26858328 PMC5865534

[46] Shafrin J Sullivan J Goldman DP Gill TM. The association between observed mobility and quality of life in the near elderly. PLoS ONE. (2017) 12:e0182920. 10.1371/journal.pone.0182920 28827806 PMC5572211

[47] Jones SE Maddocks M Kon SS Canavan JL Nolan CM Clark AL. Sarcopenia in COPD: prevalence, clinical correlates and response to pulmonary rehabilitation. Thorax. (2015) 70:213–8. 10.1136/thoraxjnl-2014-206440 25561517

[48] Watz H Waschki B Meyer T Magnussen H. Physical activity in patients with COPD. Eur Respir J. (2009) 33:262–72. 10.1183/09031936.00024608 19010994

[49] Bone AE Hepgul N Kon S Maddocks M. Sarcopenia and frailty in chronic respiratory disease. Chron Respir Dis. (2017) 14:85–99. 10.1177/1479972316679664 27923981 PMC5720213

[50] Lam K Shi Y Boscardin J Covinsky KE. Unmet need for equipment to help with bathing and toileting among older US adults. JAMA Intern Med. (2021) 181:662–70. 10.1001/jamainternmed.2021.0204 33749707 PMC7985819

[51] Hajjar ER Cafiero AC Hanlon JT. Polypharmacy in elderly patients. Am J Geriatr Pharmacother. (2007) 5:345–51. 10.1016/j.amjopharm.2007.12.002 18179993

[52] Jokanovic N Tan ECK Dooley MJ Kirkpatrick CM Bell JS. Prevalence and factors associated with polypharmacy in long-term care facilities: a systematic review. J Am Med Direct Assoc. (2015) 16:535.e1–2. 10.1016/j.jamda.2015.03.003 25869992

[53] Halli-Tierney AD Scarbrough C Carroll DG. Polypharmacy: evaluating risks and deprescribing. AFP. (2019) 100:32–8. 31259501

[54] Williams BC Fitzgerald JT. Brief report: brief instrument to assess geriatrics knowledge of surgical and medical subspecialty house officers. J Gen Intern Med. (2006) 21:490–3. 10.1111/j.1525-1497.2006.00433.x 16704394 PMC1484789

[55] Ko Y Lin SJ Salmon JW Bron MS. The impact of urinary incontinence on quality of life of the elderly. Am J Manag Care. (2005) 11 (4 Suppl):S103–11. 16161383

[56] Brown JS Vittinghoff E Wyman JF Stone KL Nevitt MC Ensrud KE. Urinary incontinence: does it increase risk for falls and fractures? Study of osteoporotic fractures research group. J Am Geriatr Soc. (2000) 48:721–5. 10.1111/j.1532-5415.2000.tb04744.x 10894308

[57] Rusanen M Ngandu T Laatikainen T Tuomilehto J Soininen H Kivipelto M. Chronic obstructive pulmonary disease and asthma and the risk of mild cognitive impairment and dementia: a population based CAIDE study. Curr Alzheimer Res. (2013) 10:549–55. 10.2174/1567205011310050011 23566344

[58] Singh B Mielke MM Parsaik AK Cha RH Roberts RO Scanlon PD. A prospective study of chronic obstructive pulmonary disease and the risk for mild cognitive impairment. JAMA Neurol. (2014) 71:581–8. 10.1001/jamaneurol.2014.94 24637951 PMC4020948

[59] CDC. BRFSS. (2022). Available online at: https://www.cdc.gov/brfss/index.html (accessed January 8, 2022).

[60] Bandeen-Roche K Seplaki CL Huang J Buta B Kalyani RR Varadhan R. Frailty in older adults: a nationally representative profile in the United States. J Gerontol A Biol Sci Med Sci. (2015) 70:1427–34. 10.1093/gerona/glv133 26297656 PMC4723664

[61] Global Initiative for Chronic Obstructive Lung Disease. Global Strategy for the Diagnosis, Management Prevention of Chronic Obstructive Pulmonary Disease. (2020). Available online at: https://goldcopd.org/wp-content/uploads/2019/12/GOLD-2020-FINAL-ver1.2-03Dec19_WMV.pdf

[62] ABMS Board Certification Report (2018-2019). Available online at: https://www.abms.org/wp-content/uploads/2020/11/abms-board-certification-report-2018-2019.pdf

[63] Older, People Need Geriatricians. Where Will They Come From? The New York Times. Available online at: https://www.nytimes.com/2020/01/03/health/geriatricians-shortage.html (accessed January 11, 2022).

[64] Extermann M Aapro M Bernabei R Cohen HJ Droz JP Lichtman S. Use of comprehensive geriatric assessment in older cancer patients:: recommendations from the task force on CGA of the international society of geriatric oncology (SIOG). Crit Rev Oncol Hematol. (2005) 55:241–52. 10.1016/j.critrevonc.2005.06.003 16084735

[65] Hsu T. Educational initiatives in geriatric oncology—who, why, and how? J Geriatr Oncol. (2016) 7:390–6. 10.1016/j.jgo.2016.07.013 27567256

[66] Fulmer T Mate KS Berman A. The age-friendly health system imperative. J Am Geriatr Sock. (2018) 66:22–4. 10.1111/jgs.15076 28876455

