# Supplementary material for: Beyond the Lung: Geriatric Conditions Afflict Community-Dwelling Older Adults With Self-Reported Chronic Obstructive Pulmonary Disease
Source: Front Med (Lausanne). 2022 Feb 14;9:814606. doi: 10.3389/fmed.2022.814606 (PMC8884078; doi:10.3389/fmed.2022.814606)
Supplement: Supplementary file 1 [file Data_Sheet_1.pdf]

**Online data supplement**

**Methods**

*Geriatric Conditions*

Geriatric conditions assessed were multimorbidity, functional disability, impaired physical function (slow timed up and go performance time), extreme low physical activity, modified frailty assessment, falls, polypharmacy, urinary incontinence. These measures were evaluated as follows:

*Multimorbidity:* Comorbidity data (asthma, heart failure, myocardial infarction history, diabetes, stroke history, cancer history and arthritis) were self-reported. Respondents were asked if a medical doctor had ever told them that they had [CONDITION]. We constructed a modified Charlson co-morbidity index, as previously described in the NSHAP data set based on the original index of 19 weighted conditions, predictive of mortality [1–3], in which co-morbidities were added with varying weights as follows: 1 point assigned to history of myocardial infarction, gastric ulcer disease, congestive heart failure, peripheral vascular disease, arthritis, dementia, asthma, and stroke; 1.5 points assigned to diabetes, 2 points assigned to liver disease, leukemia, lymphoma, renal disease, and cancer history; and 6 points assigned to metastatic cancer. COPD was removed from the score. **Possible score** ranged from 0-25.5 where a 0 score indicates no co-morbid conditions and 25.5 indicates all co-morbid conditions included.

*Functional disability:* Difficulty performing seven activities of daily living (ADL) were assessed by self-report. ADL disability was defined as any difficulty performing at least one ADL. ADLs assessed were walking a block, walking across the room,

independently dressing, independently bathing, independently eating, transferring out of bed, and independently toileting. Gait speed was assessed using the Timed Up and Go (TUG) test.[4] Slow TUG speed was defined as a Timed Up and Go test greater than 10 seconds, or being wheelchair-bound. Extreme low self-reported physical activity was defined as < once a month of moderate to vigorous activity on average.

*Frailty:* an abbreviated frailty scale was created from three measures: 1) self-reported exhaustion, 2) self-reported physical activity, and 3) Timed Up and Go speed. Self-reported exhaustion was defined by an answer of “occasionally” or “most of the time” on one or both questions from the Center for Epidemiologic Studies-Depression: 1) feeling that everything was an effort and 2) could not get going. Self-reported low physical activity was defined as physical activity 1-3 times per month or less. TUG speed was defined as slow if the time was greater than 10 seconds. Each measure was assigned one point and frailty was defined as a score of 3. Non-frailty was defined as a score less than 3. Round 1 did not assess weight loss or handgrip strength, and TUG speed not gait speed was measured, so this frailty scale is a modification from the classic phenotypic frailty criteria.[5,6]

*Medications/Polypharmacy:* Home interviewers recorded each medication taken by the respondents, and each medication was then categorized. The prevalence of high-risk Beers criteria medications[7] are reported, including anti-histamines (sedating and non-sedating), anticholinergics, benzodiazepines, anti-psychotics, anxiolytics/sedatives, tricyclic antidepressants, muscle relaxants, anti-arrhythmics, COX-2 inhibitors, and narcotics. Interviewers counted and recorded each respondent’s medications.[8] Polypharmacy has been defined variably. We defined moderate

polypharmacy as four medications or more and severe polypharmacy as ten medications or more. The proportion of individuals with polypharmacy is reported. *Falls:* Respondents were asked, “Have you fallen in the last 12 months?” The proportion of individuals answering yes to this question is reported.

*Urinary incontinence:* Respondents were asked, “Have you had urinary incontinence in the last 12 months?” The proportion of individuals answering yes to this question is reported.

*Psychosocial measures*

Social frailty measures assessed were: 1) *Frequency of social activities:* respondents were asked how frequently they socialized with friends or relatives in the last year, and rated the social activity as never, less than once a year, about once or twice a year, several times a year, about once a month, every week, or several times a week. Extreme social disengagement was categorized as frequency of social activity less than once a year (score <2). Moderate social disengagement was categorized as frequency of social activity several times a year or less (score <4).[9] 2) *Loneliness:* The NSHAP Felt Loneliness Measure (NFLM) was adapted from the Revised UCLA loneliness scale. [10] Respondents were asked if they: 1) lack companionship, 2) feel left out, 3) feel isolated. Respondents rated these questions as hardly ever/never, some of the time, or often. The maximum score was 6, with higher scores indicating increased loneliness. We report the percentage of respondents with an NFLM score ≥ 1.

Cognitive and affective measures assessed were: 1) *Cognitive impairment:* cognition was assessed using the Short Portable Mental Status Questionnaire (SPMSQ), which is a 10-item assessment that assesses items including orientation,

current president and digit subtraction. A score less than 6 defines moderate cognitive impairment. [11,12] 2) *Depressive symptoms:* The NSHAP Depressive Symptoms Measure (NDSM) was adapted from the Center for the Epidemiologic Studies Depression (CES-D) Scale. The NDSM quantifies the frequency of eleven items during the past week, selected from the original 20-item CES-D scale.[10] We report the percentage of respondents with an NDSM score greater than or equal to 9, representing significant depressive symptoms.

**References**


1. Vasilopoulos T, Kotwal A, Huisingh-Scheetz MJ, Waite LJ, McClintock MK, Dale W. Comorbidity and Chronic Conditions in the National Social Life, Health and Aging Project (NSHAP), Wave 2. *The Journals of Gerontology: Series B*. 2014;69(Suppl_2):S154-S165. doi:10.1093/geronb/gbu025

2. Charlson ME, Pompei P, Ales KL, MacKenzie CR. A new method of classifying prognostic comorbidity in longitudinal studies: development and validation. *J Chronic Dis*. 1987;40(5):373-383. doi:10.1016/0021-9681(87)90171-8

3. Sr W, G PK, Sa L. Measures of chronic conditions and diseases associated with aging in the national social life, health, and aging project. *J Gerontol B Psychol Sci Soc Sci*. 2009;64 Suppl 1:i67-75. doi:10.1093/geronb/gbn015

4. Podsiadlo D, Richardson S. The timed “Up & Go”: a test of basic functional mobility for frail elderly persons. *J Am Geriatr Soc*. 1991;39(2):142-148. doi:10.1111/j.1532-5415.1991.tb01616.x

5. Fried LP, Tangen CM, Walston J, et al. Frailty in older adults evidence for a phenotype. *The Journals of Gerontology Series A: Biological Sciences and Medical Sciences*. 2001;56(3):M146-M157.

6. Huisingh-Scheetz M, Kocherginsky M, Schumm PL, et al. Geriatric Syndromes and Functional Status in NSHAP: Rationale, Measurement, and Preliminary Findings. *J Gerontol B Psychol Sci Soc Sci*. 2014;69(Suppl 2):S177-S190. doi:10.1093/geronb/gbu091

7. American Geriatrics Society 2019 Updated AGS Beers Criteria® for Potentially Inappropriate Medication Use in Older Adults - - 2019 - Journal of the American Geriatrics Society - Wiley Online Library. Accessed January 20, 2021. https://agsjournals.onlinelibrary.wiley.com/doi/full/10.1111/jgs.15767

8. Qato DM, Schumm LP, Johnson M, Mihai A, Lindau ST. Medication Data Collection and Coding in a Home-Based Survey of Older Adults. *J Gerontol B Psychol Sci Soc Sci*. 2009;64B(Suppl 1):i86-i93. doi:10.1093/geronb/gbp036

9. Waite LJ, Duvoisin R, Kotwal AA. Social Health in the National Social Life, Health, and Aging Project. *J Gerontol B Psychol Sci Soc Sci*. 2021;76(Supplement_3):S251-S265. doi:10.1093/geronb/gbab138

10. Payne C, Hedberg EC, Kozloski M, Dale W, McClintock MK. Using and Interpreting Mental Health Measures in the National Social Life, Health, and Aging Project. *J Gerontol B Psychol Sci Soc Sci*. 2014;69(Suppl 2):S99-S116. doi:10.1093/geronb/gbu100

11. Pfeiffer E. A short portable mental status questionnaire for the assessment of organic brain deficit in elderly patients. *J Am Geriatr Soc*. 1975;23(10):433-441. doi:10.1111/j.1532-5415.1975.tb00927.x

12. Laumann EO, Leitsch SA, Waite LJ. Elder Mistreatment in the United States: Prevalence Estimates From a Nationally Representative Study. *The Journals of Gerontology: Series B*. 2008;63(4):S248-S254. doi:10.1093/geronb/63.4.S248
